# Experimental Research for the Establishment of the Optimal Forging and Heat Treatment Technical Parameters for Special Purpose Forged Semi-Finishes

**DOI:** 10.3390/ma16062432

**Published:** 2023-03-18

**Authors:** Nicolae Constantin, Adrian Ioana, Valentina Caloian, Valeriu Rucai, Cristian Dobrescu, Alexandra Istrate, Vili Pasare

**Affiliations:** 1 Materials Science and Engineering Faculty, University Politehnica of Bucharest, 060042 Bucharest, Romania; 2 Engineering and Management of Metallic Materials Obtaining Department, Science and Engineering Materials Faculty, University Politehnica of Bucharest, 060042 Bucharest, Romania

**Keywords:** forging, heat treatment, steel, tempering, resilience, plasticity

## Abstract

The authors present in this paper the experimental results and conclusions obtained after conducting a comparative study on three samples of forged semi-finished products from the steel brands 10CrMo9-10, 25CrMo4, and 42CrMo4. These are common heat-resistant alloy steels used in various industries nationally and internationally. This study aimed to test under the same identical experimental conditions of forging and heat treatment of three samples made of three different brands of steels 10CrMo9-10, 25CrMo4, and 42CrMo4. Analyzing the experimental results obtained, it can be seen for which of the three brands of tested steels the best forging and heat treatment parameters are obtained. Following experimental research, the best material was determined by analyzing the results obtained for the mechanical characteristics (tensile tests according to DNVGL-RP0034-SFC2 and NACE MR0175–hardness 207-235 HBW) and austenitic grain size. The authors determined that among the three types of steels analyzed, 10CrMo9-10 best meets the imposed requirements. This statement is in view of the comparative analysis of the results of experimental research.

## 1. Introduction

Production capacity utilization shall be calculated monthly based on gross steel production and information on the available capacity existing in the World Steel Association database [1,2,3,4].

The information submitted by the World Steel Association is based on publicly available data, updated twice a year, and verified by members of the World Steel Association [5,6]. The presence above certain limits of non-metallic inclusions in solidified steel ingots causes essential changes in their plasticity and hot deformation capacity due to the formation of structural discontinuities [7,8]. The mechanical properties of structural constituents are different and, therefore, during plastic deformation processes, do not deform evenly [9,10,11]. The interaction between the various deformed constituents leads to the appearance of tensions that can eventually lead to cracks or ruptures. The greater the difference between the mechanical properties of the various constituents, the greater the tendency to form discontinuities in the metal matrix [9].

The presence beyond certain limits of non-metallic inclusions in solidified steel ingots causes essential changes in their plasticity and thermic deformation capacity due to the formation of structural discontinuities [7,8,9,10,11]. Powdery and small ferrous waste resulting in different phases of industrial processes (in most cases steel) represents an intrinsic value, which is determined by the ferrous content (chemically bound iron, sometimes metallic) that can properly replace the raw material, namely iron ore/cast iron/scrap iron, in steel processes [6,7,8].

Since studying the absolute plastic properties of non-metallic inclusions is difficult to carry out, most experimental research sought to study the ability to deform non-metallic inclusions in the metal matrix, using relative indices comparing the plastic properties of non-metallic inclusions with those of steel in which they are included. To fully justify the need for experimental research, it should be mentioned that steel is currently one of the most used alloys in modern society [1,12,13,14].

Its versatility, durability, and resistance make it a popular choice for various fields: railway, machine building, naval, extractive, and chemical industries [15,16,17,18,19].

The forging of materials involves obtaining forged semi-finished in several successive stages and with a certain accepted deformation.

After applying the secondary heat treatment, an improved structure appears, which is no longer prone to cracks or the appearance of intergranular inclusions but is more fragile than the one obtained without secondary heat treatment [20,21,22,23,24].

The modeling of mechanical characteristics depends on the type of technological flow operations: free forging, primary TT, and secondary TT, but also on the chemical composition and the alloying elements of the steels used [25,26,27,28]. The novelty of the work is the performance of experimental research on samples of three different brands of steel, 10CrMo9-10, 25CrMo4, and 42CrMo4, subjected to forging and heat treatments under the same experimental conditions.

The research aims to find the best relationship between the resulting mechanical characteristics, heat treatment parameters, and the requirements of standards and international certification bodies.

Another novelty element is the exact establishment of a technology for the execution of a semi-finished product from 10CrMo9-10 with the best ratio of mechanical characteristics capable of meeting the conditions of international certification DNVGL-RP-0034-SFC2 and NACE MR0175 (maximum 22–23 HRC).

## 2. Materials and Method

### 2.1. Materials

To establish the best forging and heat treatment parameters of some forged semi-finished products, the authors performed comparative experimental research on 3 samples of forged semi-finished products from the steel brands 10CrMo9-10, 25CrMo4, and 42CrMo4.

The 10CrMo9-10 alloy steel specified in EN 10028-2 has good anti-corrosion properties and provides excellent performance with a higher Cr and Mo content that provides higher heat and corrosion resistance than 13CrMo4-5 and 16Mo3.

10CrMo9-10 is a high-temperature steel for pressure vessels and the transportation of petroleum products [29,30,31,32]. The chemical composition of the 10CrMo9-10 steel used in our own research is presented in Table 1.

25CrMo4 is a low-alloy steel that contains chromium and molybdenum as the main alloying elements. It is a versatile alloy with good resistance to atmospheric corrosion, strength, weldability, and processing characteristics. This steel is mainly used in the energy industry because it can be subjected to high temperatures, it has creep resistance.

Made parts: flanges, caps, nuts, turbines, toothed shafts. The chemical composition of the 25CrMo4 steel used in our own research is presented in Table 2.

The 42CrMo4 class steels are successful in the car construction industry, being used in the manufacture of high-resistance parts such as compressors, turbines, and working elements of heavy surface equipment. Steel has reduced weldability due to the high sensitivity to cracking. The chemical composition of the 42CrMo4steel used in our own research is presented in Table 3.

### 2.2. Method

Forging is done between 850–1150 °C.

The stages required for the forging process were:

- ingot forging D = 1160 mm in a square 750 × 750 × 1 (minimum degree of deformation 1.9:1).
- cutting the foot of the ingot and the semi-finished product.
- removing the semi-finished product (starting from dimensions 750 × 750 × 2119);
- adjusting the semi-finished product to the forging size.
- notching and stretching the semi-finished product for a sizing step.
- Type of heat treatment:
- Tempering: 820–860 °C/oil
- Recovery: 540–580 °C/air

Description of the heat treatment technologies applied to the studied semi-finished products.

The thermal treatments of normalization, hardening, and recovery have been carried out to obtain the quality of the analyzed semi-finished products.

A martensitic structure is obtained by hardening. Cooling is carried out at a comparatively higher speed than the steel quid speed.

The heat treatment of tempering applied to products which are called and consist of heating and maintenance at temperatures set below the transformation line so that structural conditions and voltages close to the equilibrium state are achieved, with the aim of eliminating or reducing stresses and fragility, improving tension, and reducing the hardness.

**Figure 1:**
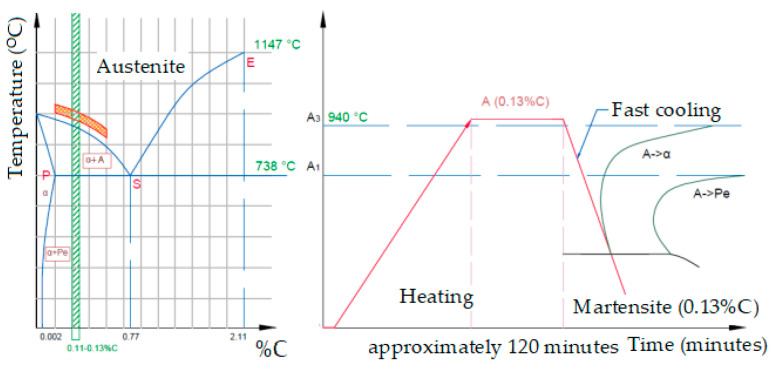
The heating range and the cycle of operations for tempering the semi-finished products [1].

**Figure 2:**
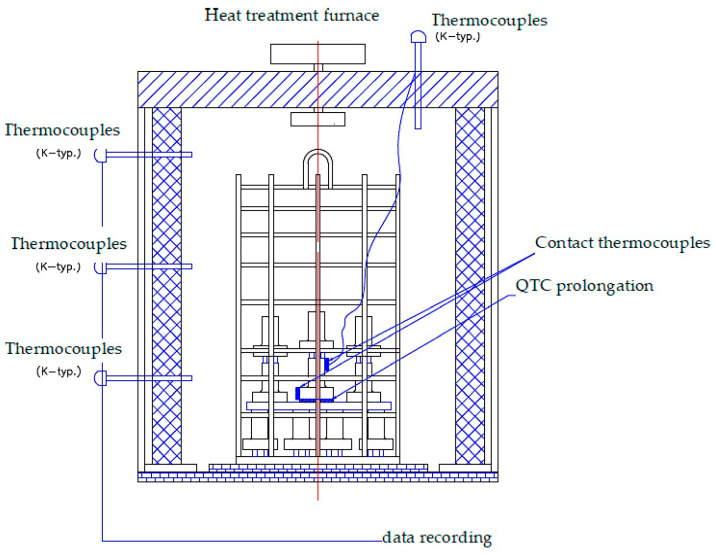
The heat treatment furnace [1].

Figure 1 shows the heating range and the cycle of operations for tempering the semi-finished products, and Figure 2 shows the diagram of the heat treatment furnace used.

#### 2.2.1. Description of Mechanical Shock Tests—Resilience

The determination shall be made by applying a shock load from a height h on a test tube with a notch in U or V, denoted KCU or KCV. The test tubes shall be placed on the reassembly using the centering pliers so that the hammer strikes the face opposite the notch.

#### 2.2.2. Description of the Determination of the Size of the Austenitic Grain for Forged Semi-Manufactures

The method used to highlight the austenitic grain is the method of controlled oxidation and is done as follows:

- A flat face of the sample must be sanded, and the rest must show no signs of oxidation [9,13];
- The sample is placed in the oven with the surface sanded up and is kept for 1 h at the austenite temperature provided for each quality of material; then open the oven door and leave the sample in its door for about 10–15 s for oxidation, then place the iron in water;
- In the case of significant oxidation, the oxide adhering to the polished surface is easily removed by grinding with fine sandpaper, after which the sample is attacked with Villella reagent (1 g picric acid, 5 mL hydrochloric acid, 100 mL ethyl alcohol) [14,15,16,17,18,19]

The size index of the austenitic grain is determined on the microscope using a 100× magnification. The determination of the size index of the austenitic grain will be done by the method of comparing it with standard images.

Optical microscopy studies provide a sequence of data that attests to the influence of the chemical composition of steels on the structure but also of the changes generated by heat treatments.

In the [33] work in the list of bibliographic references, the authors demonstrated that dynamic recrystallization occurs under different hot deformation conditions, and the size of the recrystallized grains decreases with the increase of the Zener-Hollomon (Z) parameter.

The parameter Z is the deformation rate coefficient coupled with the temperature effect, with the expression Z = ε˙ exp(Q/RT) [17], where Q is the deformation activation energy (381.34 kJ·mol^−1^) [11], R is the gas constant (8.314 J·(mol·K) ^−1^) and T is the thermodynamic temperature [33].

#### 2.2.3. Description of Metallographic Analysis—Structural Determinations

The determination of the microstructure is usually made at a magnification of ×100 or ×200 to highlight any segregations and increases of ×400 or ×500 to record the structural constituents obtained after the operation of tempering and return. The sample is taken, after which it is sanded and attacked with Nital 2%. The macrostructure is determined on samples of about 15 to 20 mm cut either from a laminate, forged, or from a piece [20,21,22,23,24,25].

The sample is rectified, degreased, and then attacked with a reagent. A mixture of 85% hydrochloric acid + 10% distilled water is usually used. The duration of the attack is one hour. After 40 min from the start of the attack, 5% oxygenated water can be put in. The area shall be examined at the magnification of ×10 (visual examination).

Following experimental research on the 3 types of analyzed steels (10CrMo9-10, 25CrMo4, and 42CrMo4), we can compare the choice of the best material as a ratio of mechanical characteristics (tests according to DNVGL-RP0034-SFC2 and NACE MR0175–Dough 207-235 HBW) and the production cycle for the three samples selected from each material as follows:

- There are different materials with different chemical compositions: 10CrMo9-10 has a lower percentage of C (0.15%) and Mn (0.55%) and more significant by Cr (2.48%), Mo (1.02%) the initial amount of Fe = 95.28%; 25CrMo4 has a higher percentage of C (0.32%) and Mn (0.73%) and lower by Cr (1.07%) and Mo (0.26%) the final amount of Fe = 96.81%; 42CrMo4 has a higher percentage of C (0.42%) and Mn (0.83%) and lower by Cr (1.04%) and Mo (0.25%) the final amount of Fe = 96.84%;
- They are regulated by different standards; therefore, 10CRMO9-10 is found in EN 10222-2 as being part of the category of ferrite and martensitic steels having specific mechanical characteristics at high temperatures, and 25CrMo4 and 42CrMo4 are both regulated by EN 10250-3 as being part of the allied special steel category.

The primary heat treatment used consists of normalization at 941–968 °C, monitoring with contact thermocouples, holding for at least 1 h after reaching the equalization temperature, and cooling to the room temperature in air.

As a secondary heat treatment for the 10CrMo9-10 material, a quenching at 940 °C with a 120 min hold and cooling in water was applied, followed by tempering at 655 °C with a 180 min hold and air cooling.

### 2.3. Equipment Used for Experimental Research

The equipment used in the experimental research were a Charpy shock testing machine with a low-temperature chamber, a tensile testing machine, an electronic axial strain gauge for a tensile testing machine, Spectrometer, a metallographic optical microscope, and a Brinell hardness tester.

## 3. Results and Discussion

### 3.1. Results

#### 3.1.1. Results Obtained for the Forged Semi-Finished 10CrMo9-10

The results obtained after the mechanical tests are presented in Table 4.

Results obtained after determining the austenitic grain index are presented in Table 5.

These results were obtained under the experimental conditions presented in Table 6.

**Figure 3:**
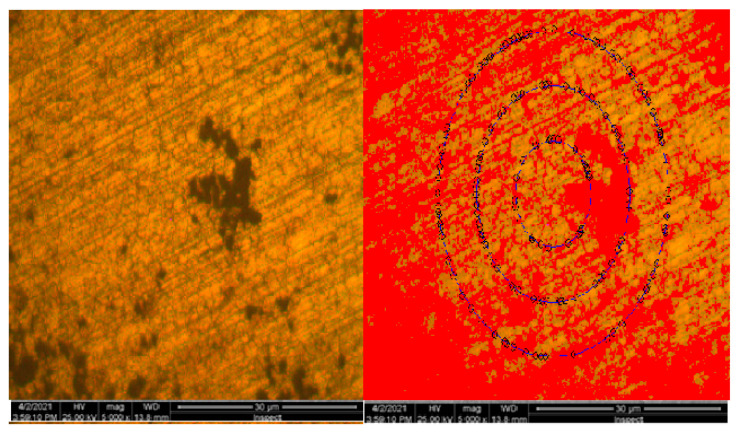
Microscopic appearance of austenitic grains for the forged semi-finished product 10CrMo9-10 [1].

Microscopic appearance of austenitic grains for the forged semi-finished product 10CrMo9-10 is presented in Figure 3.

**Figure 4:**
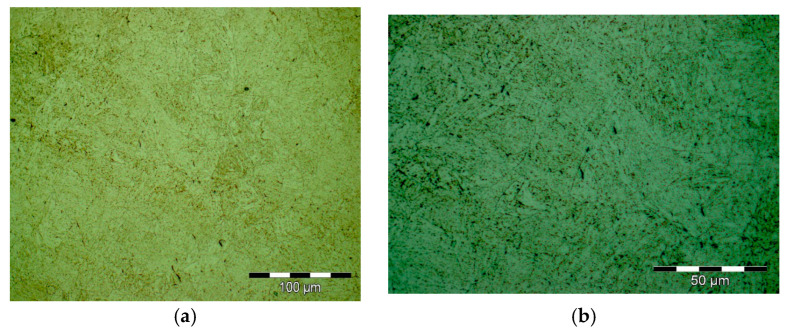
Optical microscopy images from secondary heat treatment; 500× magnification (**a**) 1000× (**b**).

Results obtained for structural investigations by optical microscopy are presented in Figure 4.

**Figure 5:**
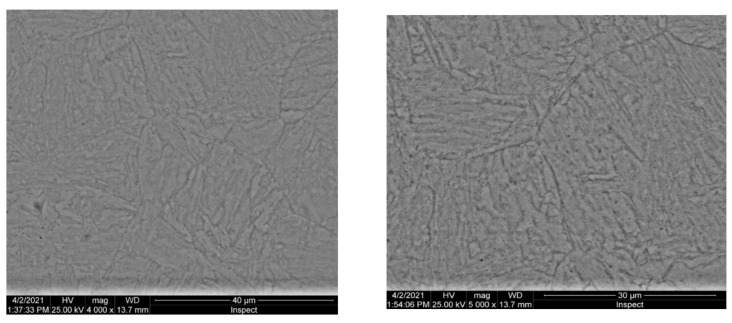
Electronic scanning microscopy for 10CrMo9-10 sample.

Results obtained from the structural investigation performed by electron scanning microscopy (SEM) are presented in Figure 5.

Optical microscopy studies provide a sequence of data that attests to the influence of the chemical composition of steel on the structure, as well as the changes generated by thermal treatments.

**Figure 6:**
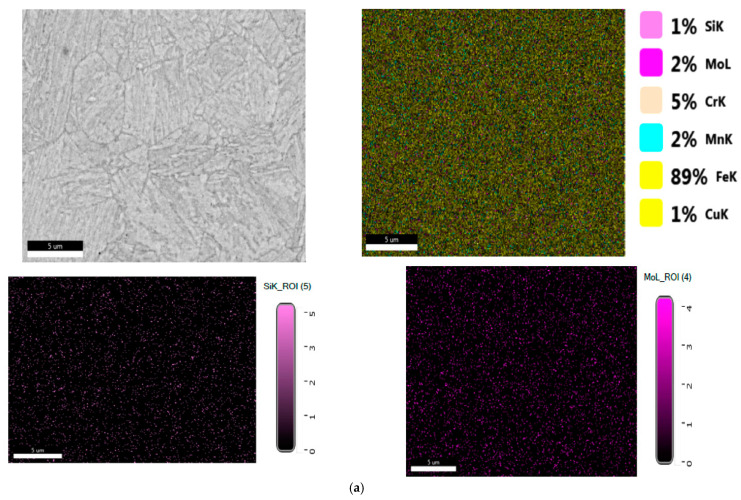

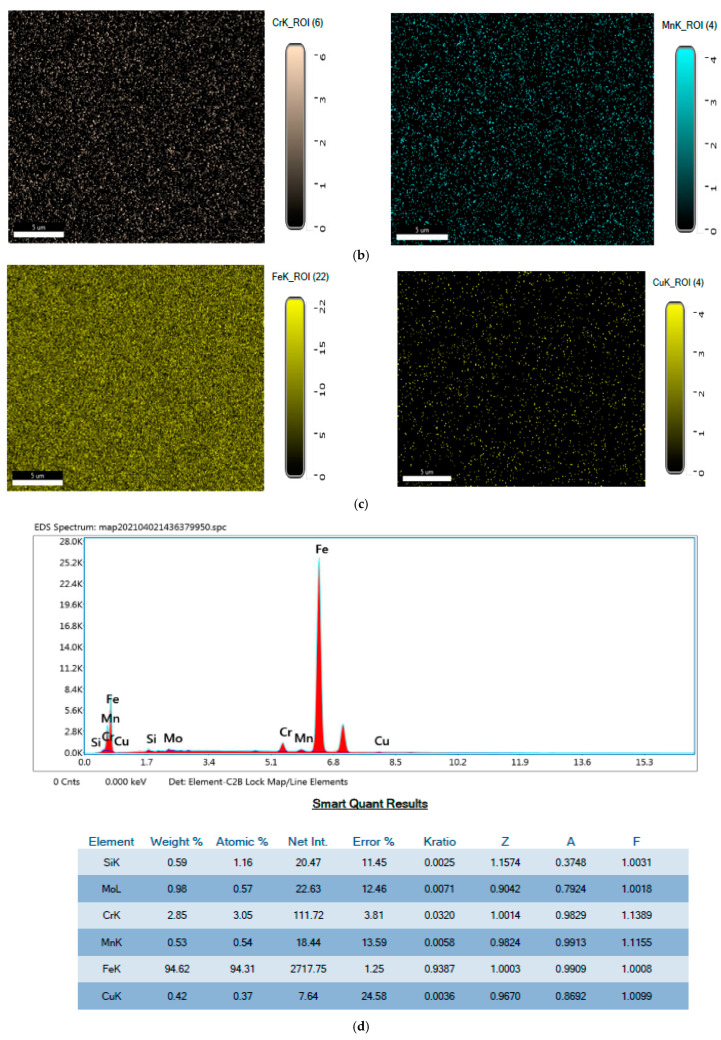
(**a**) EDS spectrum for the Si element from the 10CrMo9-10 steel sample. (**b**) EDS spectrum for the Cr element from the 10CrMo9-10 steel sample. (**c**) EDS spectrum for the Fe element from the 10CrMo9-10 steel sample. (**d**) The spot chemical analysis in the selected area for the 10CrMo9-10 steel sample.

Results obtained for structural investigation of energy dispersion spectrometry (EDS, are presented in Figure 6.

#### 3.1.2. Results Obtained for the Forged Semi-Finished 25CrMo4

The results obtained after the mechanical tests are presented in Table 7.

Results obtained after determining the austenitic grain index are presented in Table 8.

These results were obtained under the experimental conditions presented in Table 9.

**Figure 7:**
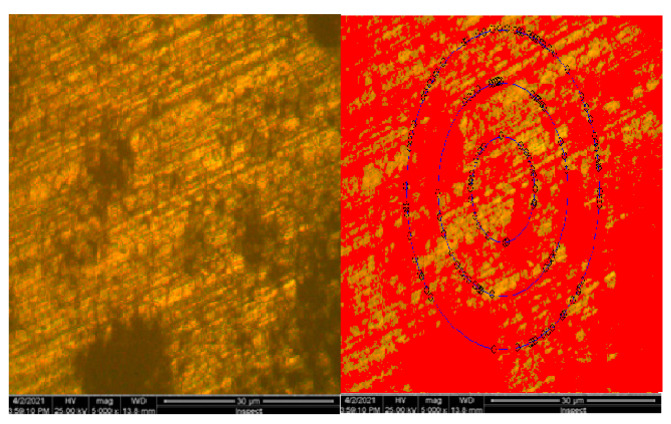
Microscopic appearance of austenitic grains for the forged semi-finished product 25CrMo4 [1].

Microscopic appearance of austenitic grains for the forged semi-finished product 10CrMo9-10 is presented in Figure 7.

**Figure 8:**
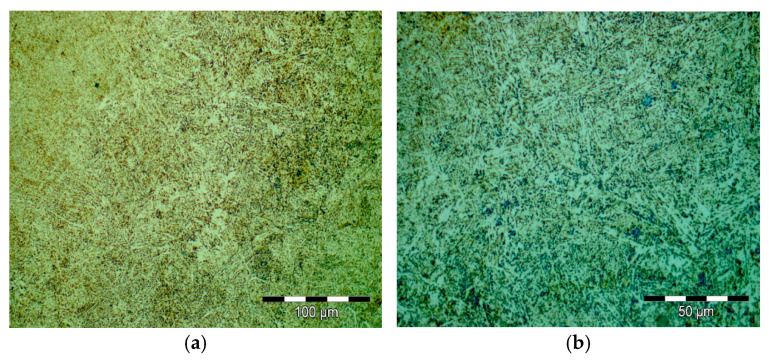
Optical microscopy images from secondary heat treatment; 500× magnification (**a**) 1000× (**b**).

Results obtained for structural investigations by optical microscopy are presented in Figure 8.

**Figure 9:**
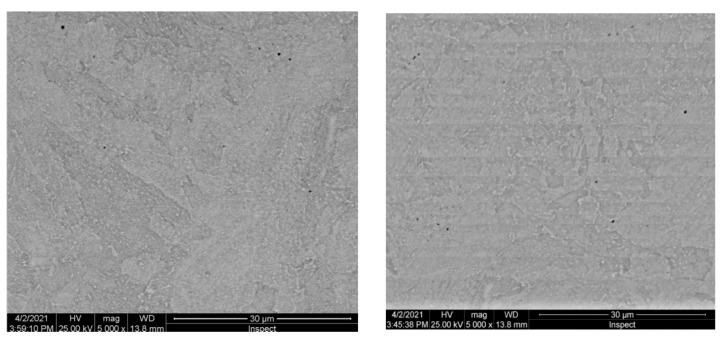
Electronic scanning microscopy for 25CrMo4 steel sample.

Results obtained from the structural investigation performed by electronic scavenging microscopy (SEM) are presented in Figure 9.

Optical microscopy studies provide a sequence of data that attests to the influence of the chemical composition of steel on the structure, as well as the changes generated by thermal treatments.

**Figure 10:**
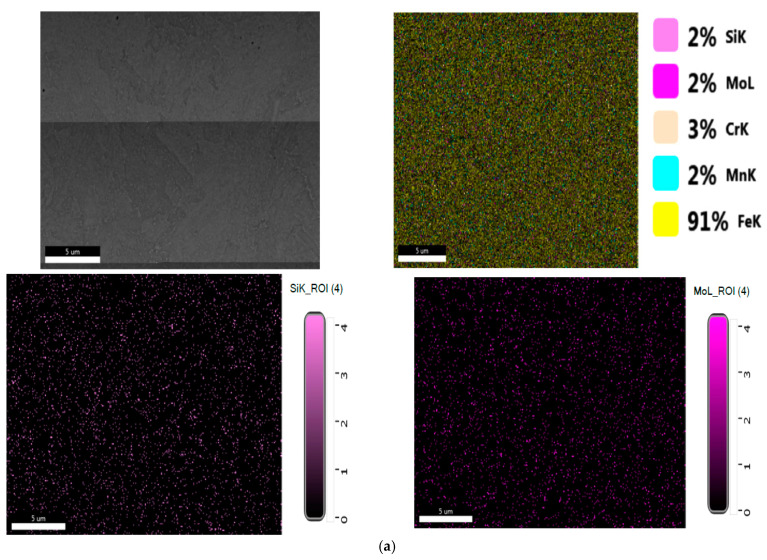

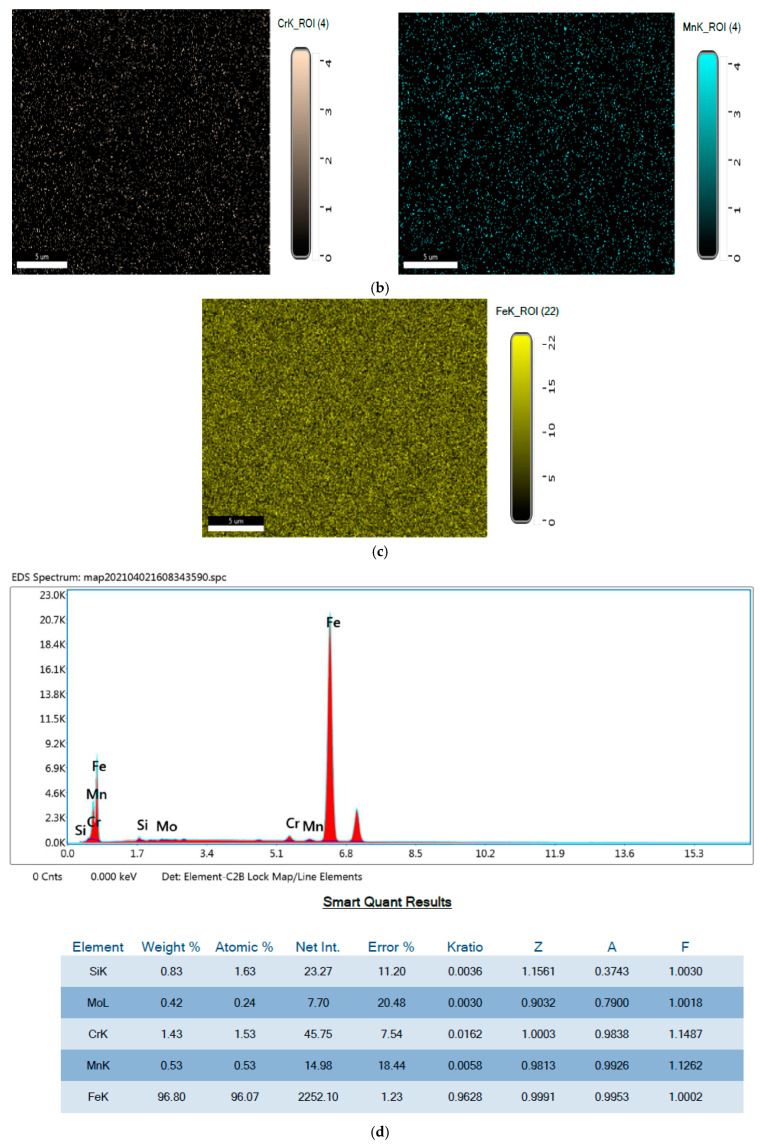
(**a**) EDS spectrum for the Si element from the 25CrMo4 steel sample. (**b**) EDS spectrum for the Cr element from the 25CrMo4 steel sample. (**c**) EDS spectrum for the Fe element from the 25CrMo4 steel sample. (**d**) The spot chemical analysis in the selected area for the 25CrMo4 sample.

Results obtained for structural investigation of energy dispersion spectrometry (EDS, are presented in Figure 10.

#### 3.1.3. Results Obtained for the Forged Semi-Finished 42CrMo4

The results obtained after the mechanical tests are presented in Table 10.

Results obtained after determining the austenitic grain index are presented in Table 11.

These results were obtained under the experimental conditions presented in Table 12.

**Figure 11:**
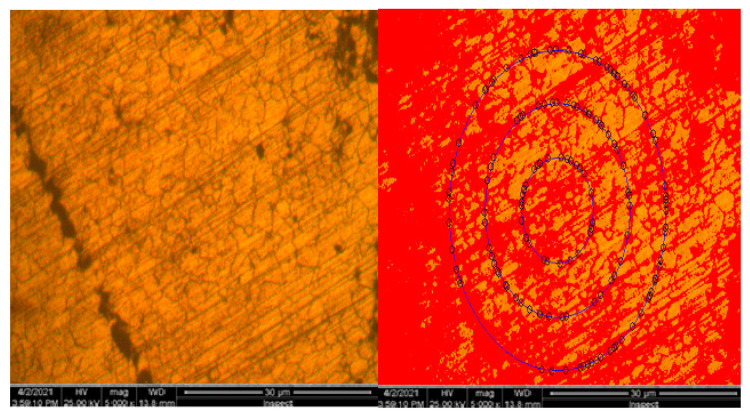
Microscopic appearance of austenitic grains for the forged semi-finished product 42CrMo4 [1].

Microscopic appearance of austenitic grains for the forged semi-finished product 42CrMo4 is presented in Figure 11.

**Figure 12:**
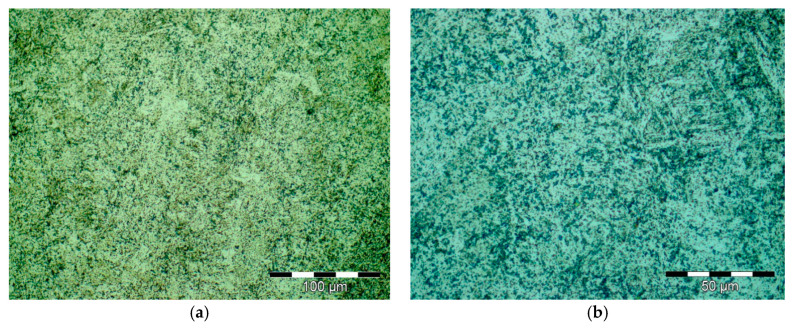
Optical microscopy images from secondary heat treatment; 500× magnification (**a**) 1000× (**b**).

Results obtained for structural investigations by optical microscopy are presented in Figure 12.

**Figure 13:**
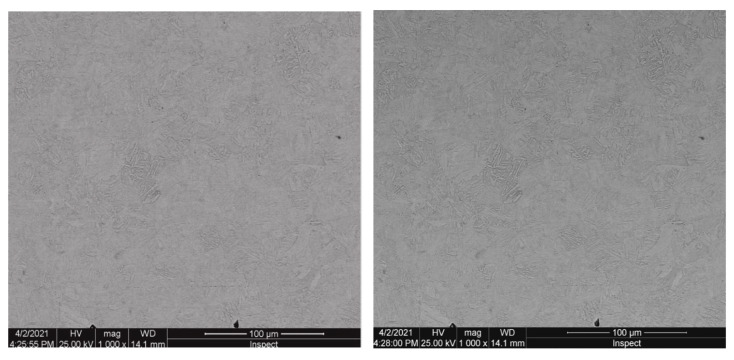
Electronic scanning microscopy for 42CrMo4 sample.

Results obtained from the structural investigation performed by electronic scavenging microscopy (SEM) are presented in Figure 13.

Optical microscopy studies provide a sequence of data that attests to the influence of the chemical composition of steel on the structure, as well as the changes generated by thermal treatments.

**Figure 14:**
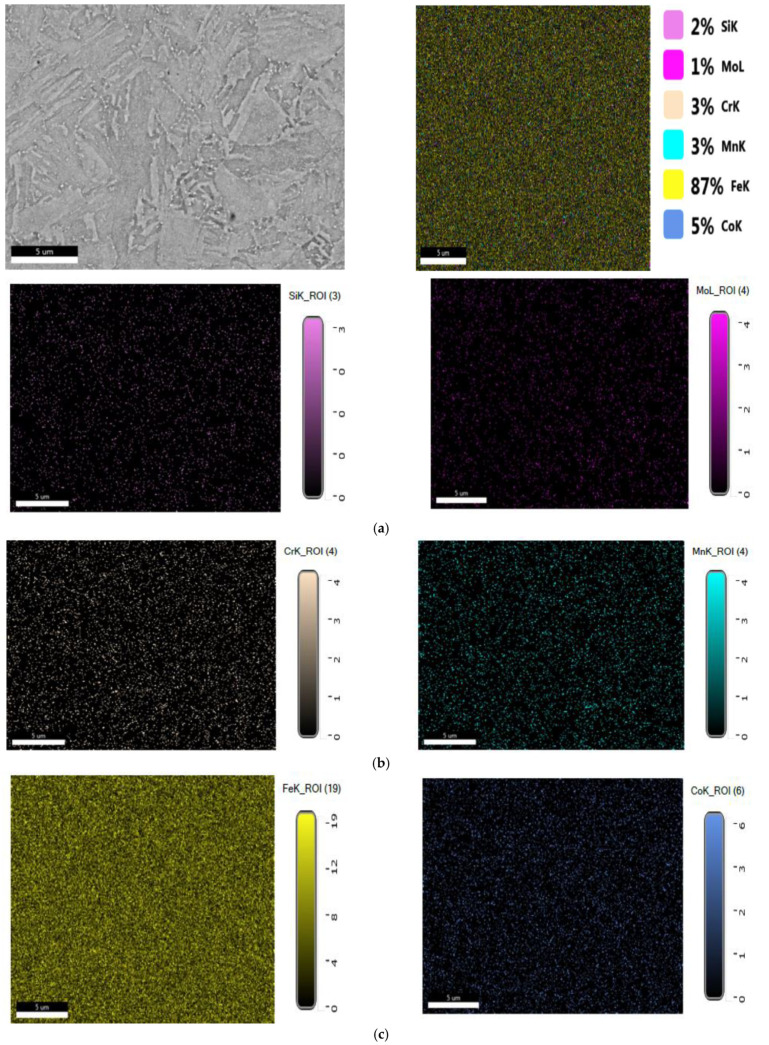

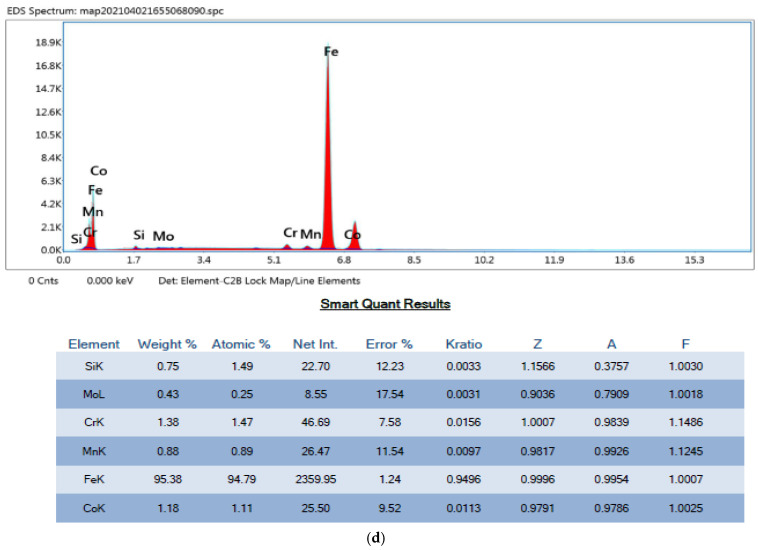
(**a**) EDS spectrum for the Si element from the 42CrMo4 steel sample. (**b**) EDS spectrum for the Cr element from the 42CrMo4 steel sample. (**c**) EDS spectrum for the Fe element from the 42CrMo4 steel sample. (**d**) The spot chemical analysis in the selected area for 42CrMo4sample.

Results obtained for structural investigation of energy dispersion spectrometry (EDS, are presented in Figure 14.

### 3.2. Discussion

To choose the best forging and heat treatment parameters, a comparative study was carried out for 3 analyzed steel samples, 10CrMo9-10, 25CrMo4, and 42CrMo4.

They are the usual heat-resistant alloy steel used in various national and international industries with specific applications at high temperatures and breaking energy down to a temperature of −80 °C.

This comparative study aims to establish the best forging parameters and thermal treatment to obtain the best ratio between the mechanical characteristics obtained and the parameters of the forged semi-finance cycle.

The primary heat treatment used in the presented technology for the 10CRMo9-10 material was normalization at 941–968 °C, monitoring with contact thermocouples, holding for at least 1 h after reaching the equalization temperature, and cooling to room temperature in air.

As a secondary heat treatment for this manufacturing technology, a quenching at 940 °C with a 120 min hold and cooling in water was applied to the 10CRMo9-10 material, followed by a return to 655 °C, a 180 min hold and air cooling.

As the parameters of the forging process and thermal treatments are relatively similar, as a maintenance interval, the highest values we find are 25CrMo4 (300 min for quenching and 600 min at high return), 42CrMo4 (120 min for hardening and 120 min at Return), and 10CrMo9-10 (60 min for both hardening and reversing).

For the material 42CrMo4 (steel alloyed with Cr and Mo), the same parameters were used for 10CrMo9-10: forging temperature 1150 °C (forging end temperature above 850 °C), ingot 25 tons with the application of primary heat treatment of normalization.

These results were obtained under the experimental conditions presented in Table 13.

After applying the secondary heat treatment, an improved structure favorable for mechanical processing appears; it is no longer prone to cracks or the appearance of intergranular inclusions, harder but a little more fragile than the one obtained from 10CrMo9-10 (following the application of the same MPS technology).

The same technology was applied to a 25CrMo4 semi-finished product: 1150 °C as forging temperature and finally, secondary heat treatment at 860 °C with maintenance after 60 min equalization and cooling in water followed by a return to 550 °C with 120 min maintenance and cooling in the air.

The mechanical characteristics obtained are relatively close to the highest.

The elongation and the cooking have little lower values as the next 10CrMo4; thus, a = 24% and z = 66%. The breaking energy is identical to all three analyzed samples. As a characteristic of steels involving an analysis of Rm and RP0.2, it represents ductility (ratio between rm/rp0.2). In the case of steel 10CrMo9-10 ductile = rm/rp0.2 = 1.21; for 25CrMo4 ductility = 1.25; for 42CrMo4 ductility = 1.27.

Considering all these aspects, it can be accepted that the 42CrMo4 sample has superior characteristics to the other steel brands tested.

The 10CrMo9-10 steel grade can be considered for use at high temperatures, which has lower forging and heat treatment costs than the other tested steel brands.

The results of the conducted research recommend the large-scale use of semi-finished products made of steel brand 10CrMo9-10.

The following conclusions can be drawn from the tests carried out on 42CrMo4 and 25CrMo4 steels:

The DNV-GL-ST-F101 is widely used in the field of metallurgy, especially forged semi-finished products in the field of extraction, natural gas, petroleum, and maritime. The procedure for issuing a certificate of material with international recognition of DNV-G type 3.2 involves meeting essential criteria both in the fields of forging and thermal treatment.

## 4. Conclusions

As a result of the forging process, it was observed that the strength and the deformation velocity (vectorial quantity) decrease with the increase in temperature.

After applying the secondary heat treatment, an improved structure favorable for mechanical processing appears; it is no longer prone to cracks or the appearance of intergranular inclusions.

The variation of austenitic grain size depends on the concentration of alloying elements and temperature; for the benchmark forged at 1150 °C from 42CrMo4, a coarse structure consisting of lamellar pearlite, bainite, and ferrite in reduced proportions was found.

The final value of the mechanical characteristics depends on the type of operations in the technological flow: free forging, the type of primary heat treatment, the type of secondary heat treatment, and the chemical composition and alloying elements of the steels used.

Experimental laboratory research shows that the 42CrMo4 steel has the highest value of mechanical characteristics, and the 10CrMO9-10 steel presents the best value for plasticity.

The 42CrMo4 brand steel sample presented the best metallographic structure, forged at 1150 °C, where a rough structure consisting of lamella, bainite, and ferrite in small proportions was found.

- Both the forging technique and the types of heat treatment applied are important to meet the requirements of the evaluation norm and offer the possibility of issuing an internationally recognized quality certificate.
- The obtained results can be used to establish different technologies for the elaboration of steels from the analyzed materials at the national and international industrial levels.

## Figures and Tables

**Table 1:** Chemical composition of 10CrMo9-10 [9].

C %	Mn%	Si%	P%	S%	Cr%	Mo%	Cu%
0.08	0.40	<0.50	<0.020	<0.010	2.00	0.90	<0.30
0.14	0.80				2.50	1.10	

**Table 2:** Chemical composition of 25CrMo4 [9].

C%	Si % max	Mn%	P% max	S%	Cr%	Mo%
0.22	0.40	0.60	0.025	0.035	0.90	0.15
0.29		0.90			1.20	0.20

**Table 3:** Chemical composition of 42CrMo4 [9].

C%	Si%	Mn%	P%	S%	Cr%	Mo%	Cu%
0.38	0.10	0.60	<0.025	<0.035	0.90	0.15	<0.40
0.45	0.40	0.90			1.20	0.30	

**Table 4:** The results of the mechanical tests for 10CrMo9-10 [1].

Product						Material		HN	HN		Specification	
Forged						10CrMo9-10		91,884	11,466		ISO 6892	
Sample size (mm)						102 × 102 × 204 mm						
Mechanical characteristics												
Traction test						The impact test						Hardness
Method												
ISO 6892-1		ISO-6892-2		-	-	ISO 148-1						ISO 6506-1
Sampling mode	Specimen orientation	Sample size (mm)				Sample size			Lateral expansion			Temperature (°C)
		Ø 12.5		Ø 10		10 × 10 × 55						22
		x										
		Temperature (°C): 22				Temperature (°C): −60						
		Rp0.2 [N/mm^2^]	Rm [N/mm^2^]	A (%)	Z (%)	KV (J)	KV (J)	KV (J)	mm	mm	mm	HBW
1/4T	L	596	719	235	77	240	240	240	-	-	-	223

**Table 5:** The results were obtained after determining the austenitic grain index for the 10CrMo9-10 sample [1].

Current Number	Intercepts Number	Grain Size (mm)
1.	115	6
2.	141	7
3.	139	7
4.	120	6

**Table 6:** The experimental conditions for determining the austenitic grain index for 10CrMo9-10 [1].

Material	10CrMo9-10
Method	E112-2013
Equipment	MICROSCOPE JP-6A
Objective	×100
HT	11,466
HN	91,884

**Table 7:** The results of the mechanical tests for 25CrMo4 [1].

Product						Material		HN		HN		Specification
Forged						25CrMo4		51,185		50,531		ISO 6892
Sample size (mm)						102 × 102 × 204						
Mechanical characteristics												
Traction test						The impact test						Hardness
Method												
ISO 6892-1		ISO-6892-2		-	-	ISO 148-1						ISO 6506-1
Sampling mode	Specimen orientation	Sample size (mm)				Sample size			Lateral expansion			Temperature (°C)
		Ø 12.5		Ø 10		10 × 10 × 55						22
		x										
		Temperature (°C): 22				Temperature (°C): −60						
		Rp0.2 [N/mm^2^]	Rm [N/mm^2^]	A (%)	Z (%)	KV (J)	KV (J)	KV (J)	mm	mm	mm	HBW
1/4T	L	597	728	255	705	240	240	240	-	-	-	235

**Table 8:** The results were obtained after determining the austenitic grain index for the 25CrMo4 sample [1].

Current Number	Intercepts Number	Grain Size (mm)
1.	139	7
2.	139	7
3.	128	7
4.	123	6

**Table 9:** The experimental conditions for determining the austenitic grain index for 25CrMo4 [1].

Material	25CrMo4
Method	E112-2013
Equipment	MICROSCOPE JP-6A
Objective	×100
HT	50,531
HN	51,185

**Table 10:** The results of the mechanical tests for 42CrMo4 [1].

Product						Material		HN		HN		Specification
Forged						42CrMo4		92,083		11,606		ISO 6892
Sample size (mm)						102 × 102 × 204						
Mechanical characteristics												
Traction test						The impact test						Hardness
Method												
ISO 6892-1		ISO-6892-2		-	-	ISO 148-1						ISO 6506-1
Sampling mode	Specimen orientation	Sample size (mm)				Sample size			Lateral expansion			Temperature (°C)
		Ø 12.5		Ø 10		10 × 10 × 55						22
		x										
		Temperature (°C): 22				Temperature (°C): −60						
		Rp0.2 [N/mm^2^]	Rm [N/mm^2^]	A (%)	Z (%)	KV (J)	KV (J)	KV (J)	mm	mm	mm	HBW
1/4T	L	607	784	215	62	212	220	215	-	-	-	229

**Table 11:** The results were obtained after determining the austenitic grain index for the 42CrMo4 sample [1].

Current Number	Intercepts Number	Grain Size (mm)
1.	161	7
2.	161	7
3.	173	7
4.	165	7

**Table 12:** The experimental conditions for determining the austenitic grain index for 42CrMo4 [1].

Material	42CrMo4
Method	E112-2013
Equipment	MICROSCOPE JP-6A
Objective	×100
HT	11,606
HN	92,083

**Table 13:** The results were obtained under experimental conditions.

Steel Brands	Rm, N/mm^2^	Rp0.2, N/mm^2^	A, %	Z, %	KV	HBV
10CRMo9-10	697	577	24	76	240	217
42CrMo4	843	512	15	31	210	255

## Data Availability

Not applicable.
